# Genetic control of tomato fruit cracking through *SlPMEI27*-dependent pectin modification

**DOI:** 10.1186/s43897-025-00219-2

**Published:** 2026-05-11

**Authors:** Fanyue Meng, Haonan Qi, Zhen Ding, Peiwen Wang, Xiuling Chen, Yang Liu, Chao Gong, Mozhen Cheng, Aoxue Wang

**Affiliations:** 1 College of Horticulture and Landscape Architecture, Northeast Agricultural University, Harbin, China; 2 College of Life Sciences, Northeast Agricultural University, Harbin, China; 3 Key Laboratory of Biology and Genetic Improvement of Horticultural Crops (Northeast Region), Ministry of Agriculture, Harbin, China; 4 College of Agriculture and Biology, Shanghai Jiao Tong University, Shang Hai, China; 5 Guangdong Academy of Agricultural Sciences, Guangdong Key Laboratory for New Technology Research of Vegetables, Vegetable Research Institute, Guangzhou, 510640 China

**Keywords:** Tomato, Fruit cracking, Cell wall structure, Pectin methylation, Transcriptome

## Abstract

Fruit cracking severely affects tomato quality and yield. By utilizing genetic transformation, knockout of the *SlPMEI27* gene can enhance the activity of pectin methylesterases (PMEs). Here, by conducting an immunofluorescence analysis, we observed that the pectin methylation rate decreased by approximately 46% (versus approximately 70% in the overexpression line), while calcium-mediated pectin cross-linking enhancement increased fruit hardness by 1.8-fold. In contrast, *SlPMEI27* overexpression inhibits PME activity, resulting in highly methyl-esterified pectin. Transmission electron microscopy analysis demonstrated that *SlPMEI27* overexpression significantly reduced cell-to-cell adhesion and increased intercellular space expansion. These factors contribute significantly to susceptibility to cracking. Additional evidence, such as enzyme activity and the expression levels of related genes, shows that the interaction between SlPMEI27 and SlGAUT7 regulates pectin modification and biosynthesis. Transcriptomic analysis revealed that *SLPMEI27* is associated with altered expression of downstream cell wall hydrolases, such as pectate/pectin lyases (PL), polygalacturonases (PG), and β-galactosidases (β-Gal), as well as ripening-related transcription factors (bHLH, ERF, and MYB). This study demonstrates that *SlPMEI27* regulates cracking by directly modulating pectin methylesterification and cell wall mechanical properties. These findings establish pectin methylesterification status as a crucial determinant of cell wall integrity during fruit development, offering a theoretical foundation for breeding crack-resistant tomatoes.

**Supplementary Information:**

The online version contains supplementary material available at 10.1186/s43897-025-00219-2.

## Core

This study identifies SlPMEI27 as a key regulator of tomato fruit cracking. Its overexpression inhibits pectin methylesterase, leading to highly methylesterified pectin, reduced cell adhesion, and increased cracking. Conversely, gene knockout enhances pectin demethylesterification and calcium cross-linking, strengthening the cell wall and fruit integrity. The interaction between SlPMEI27 and SlGAUT7 synchronizes pectin modification and biosynthesis.

## Gene and accession numbers

Sequence data in this article could be retrieved from the plant genome database (https://phytozome-next.jgi.doe.gov/) under the accession numbers: *SlPMEI27*(*Solyc08g006690*), *SlGAUT7*(*Solyc01g100210*).

## Introduction

Tomato (*Solanum lycopersicum*) is a berry-type fruit vegetable consumed both fresh and processed, and it is known for its rich content of essential nutrients for human consumption. As one of the most extensively cultivated vegetable crops globally, tomato holds a crucial position in international vegetable trade (Fareed et al. 2025). With the increase in living standards, consumers are increasingly preferring fruits and vegetables with superior quality, flavor, and commercial value. Texture attributes, particularly firmness and crack resistance, substantially influence purchasing decisions, especially for premium fresh market varieties. Fruit cracking, a common physiological disorder affecting numerous horticultural crops, including tomato, sweet cherry (*Prunus avium* L.), grape (*Vitis vinifera* L.), watermelon (*Citrullus lanatus*), and blueberry (*Vaccinium corymbosum* L.) (Khadivi-Khub, 2015), substantially impacts marketability. This condition manifests most prominently during the final stages of fruit expansion and ripening, when cell wall remodeling reaches its peak activity. Surface cracking not only diminishes visual appeal but also increases susceptibility to pathogens, thereby reducing shelf life and leading to substantial economic losses (Khadivi-Khub 2015). In tomato production, postharvest losses constitute approximately 50% of the global yield annually (Thole et al. 2021). Therefore, minimizing transportation and storage losses, extending shelf life, and enhancing fruit quality are the primary objectives in horticultural crop breeding.

Fruit ripening involves several complex and interrelated processes, including modifications in texture, cell wall ultrastructure, and composition (Li et al. 2023; Liu et al. 2025; Wang and Seymour 2022). These developmental changes require precise regulation, as excessive cell wall loosening promotes cracking, while insufficient softening compromises eating quality. Cell wall composition and modification demonstrate strong correlations with fruit cracking (Domínguez et al. 2012, 2009; López-Casado et al. 2007). The plant cell wall is a dynamic and intricate structure composed of polysaccharides and proteins, primarily cellulose, hemicellulose, and pectin (Fry 2004). Recent advances in cell wall biology have revealed that both chemical composition and architectural arrangement of these components determine mechanical performance (Hu et al. 2025). Pectin, as a major structural component, interacts with other cell wall elements through covalent and noncovalent bonds, thus playing a vital role in cell wall integrity and function (Levesque-Tremblay et al. 2015). Pectin is a group of complex polysaccharides characterized by α−1,4-linked D-galacturonic acid residues (Wolf, Mouille, and Pelloux 2009). Its structural framework includes homogalacturonan (HGA), xylogalacturonan, apiogalacturonan, rhamnogalacturonan I, and rhamnogalacturonan II (Harholt et al. 2010). HGA is the most abundant pectic polysaccharide in the primary cell wall and accounts for ~ 65% of pectin. HGA is synthesized in the Golgi apparatus through HGA galacturonosyltransferases (GAUTs) (Atmodjo et al. 2011; Dai et al. 2006; Sterling et al. 2006). Given its structural prominence, HGA modifications directly influence cell wall remodeling (Wolf, Mouille, and Pelloux 2009).

The structure and function of cell wall pectin are intrinsically linked to its degree of methylesterification, which is coordinately regulated by pectin methylesterases (PMEs) and pectin methylesterase inhibitors (PMEIs) (Coutinho et al. 2003). PME (EC 3.1.1.11) catalyzes the specific demethylesterification of HGA in plant cell walls, releasing methanol and protons while generating negatively charged carboxyl groups (De-la-Peña, Badri, and Vivanco 2008). The degree and pattern of HGA methylesterification critically determine the biomechanical properties of the cell wall. When several consecutive galacturonic acid residues undergo demethylesterification, the resulting negatively charged carboxyl groups form calcium bridges with adjacent HGA molecules, resulting in the characteristic “egg-box” structure that underlies pectin gel formation (Sénéchal et al. 2014). This calcium-crosslinked, demethyl-esterified HGA increases water-binding capacity within the cell wall matrix, with hydration status serving as a key determinant of the biomechanical properties of the cell wall, such as rigidity. Notably, the strength of pectin gels shows direct correlation with apoplastic calcium ion concentration, as gel hardness decreases with calcium bridge dissociation. Conversely, partially demethyl-esterified HGA (either randomly or in block-wise patterns) can serve as substrates for pectin-degrading enzymes, including polygalacturonases (PGs) and pectate/pectin lyases (PLs) (Wormit and Usadel 2018). The activity of PMEs is further modulated by endogenous PMEIs, which form specific 1:1 noncovalent complexes with PMEs to inhibit their enzymatic activity (Coculo and Lionetti 2022; Lionetti 2015). The first PMEI was identified in kiwi fruit (*Actinidia deliciosa*), where active PMEI was detected exclusively in fully ripe fruits but not in immature specimens. Subsequent studies confirmed progressive upregulation of PMEI gene expression during kiwi fruit maturation (Ciardiello et al. 2004). Functional studies across species revealed the diverse physiological roles of PMEIs: overexpression of *OsPMEI28* in rice (*Oryza sativa*) resulted in shortened culms accompanied by reduced cell wall thickness at stem nodes (Nguyen et al. 2017), while in *Arabidopsis*, *AtPMEI10*, *AtPMEI11*, and *AtPMEI12* serve as mediators of cell wall integrity maintenance during plant immunity (Lionetti et al. 2017). The *pmei10/11/12* triple mutants exhibited substantially reduced pectin methylesterification following pathogen challenge and compromised resistance to *Botrytis cinerea* infection (Swaminathan et al. 2021).

To date, the specific regulatory mechanisms underlying *PMEI*-mediated control of fruit cracking remain unexplored. Our laboratory previously identified *PMEI27* as a candidate gene responsible for aberrant ripening phenotypes in a tomato delayed-ripening mutant (Fig. S1), which encodes a functional PMEI (Cheng et al. 2022). In the present study, we systematically investigated the role of *SlPMEI27* in tomato fruit cracking through genetic and cellular morphological approaches. Transgenic analyses demonstrated that *SlPMEI27*-overexpressing (*SlPMEI27-*OE) lines exhibited significantly higher fruit cracking incidence compared to wild-type (WT) controls, whereas *SlPMEI27*-Cas9 (*slpmei27*) lines showed markedly reduced susceptibility to cracking, thus establishing a clear functional association between SlPMEI27 activity and fruit integrity. Comprehensive cellular and biochemical characterization using transmission electron microscopy, histological sectioning, and immunofluorescence localization revealed that SlPMEI27 modulates fruit cracking through coordinated regulation of intercellular adhesion, pectin methylesterification status, and the activity of key pectin-modifying enzymes. We also evaluated the extensive effects of *SlPMEI27* on fruit ripening and cell wall metabolism through transcriptomics. These findings represent the first experimental evidence for endogenous PMEI-mediated regulation of fruit cracking and provide novel mechanistic insights into pectin methylesterification dynamics during fruit development. The obtained results substantially advance our understanding of the molecular networks regulating pectin metabolism in ripening fruits and highlight *SlPMEI27* as a critical regulator of cell wall remodeling processes associated with fruit cracking. The present investigation establishes a foundation for future studies aimed at manipulating pectin metabolism to improve fruit quality and reduce postharvest losses.

## Results

### *SlPMEI27* expression patterns and phenotypic analysis of transgenic lines

**Fig. 1:**
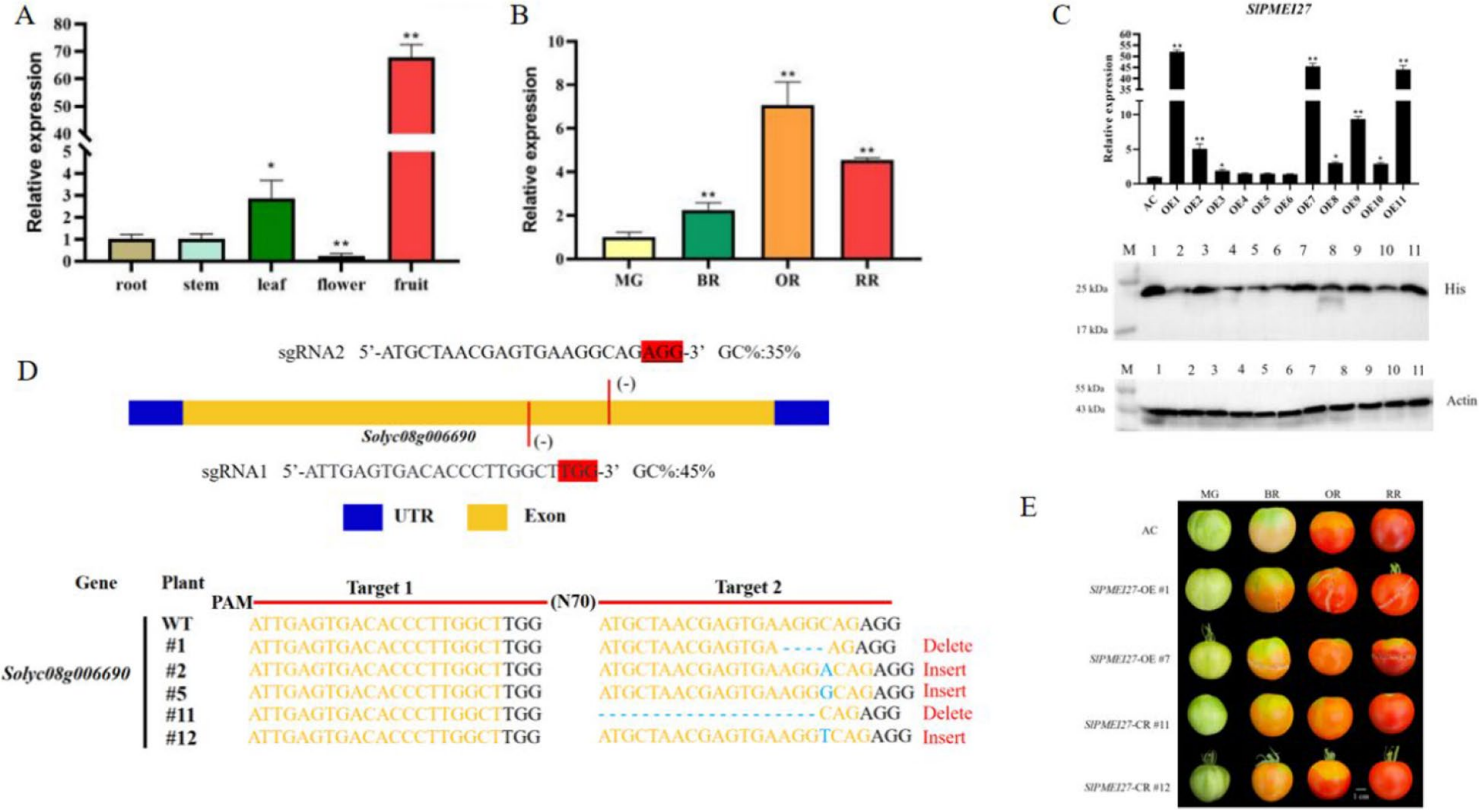
Expression analysis and transgenic characterization of *SlPMEI27* in tomato*.* **A** Tissue-specific expression of *SlPMEI27* analyzed by qPCR. **B** The expression level of the *SlPMEI27* gene during fruit ripening stages: MG, BR, OR and RR. **C** The expression level of the *SlPMEI27* gene in overexpressing tomato plants. **D** *SlPMEI27* gene knockout target design and sequencing results. **E** Phenotypic observation of transgenic plant fruit. * represents a significant difference, *P* < 0.05, ** represents a very significant difference,* P* < 0.01

Tissue-specific expression analysis revealed that *SlPMEI27* was predominantly expressed in tomato fruits, with the highest transcript levels detected in fruit tissues, followed by leaves. Minimal expression was detected in roots, stems, and flowers (Fig. 1A). During fruit development, *SlPMEI27* expression reached its peak at the OR stage, followed by a progressive decline in the RR, BR, and MG stages (Fig. 1B). To elucidate the functional role of *SlPMEI27-*OE and *slpmei27* transgenic tomato lines were generated and subsequently characterized (Fig. 1C, D). Phenotypic observations revealed that OE fruits developed severe cracking starting from the BR stage, while WT and Cas9 fruits maintained their integrity throughout development (Fig. 1E).

**Fig. 2:**
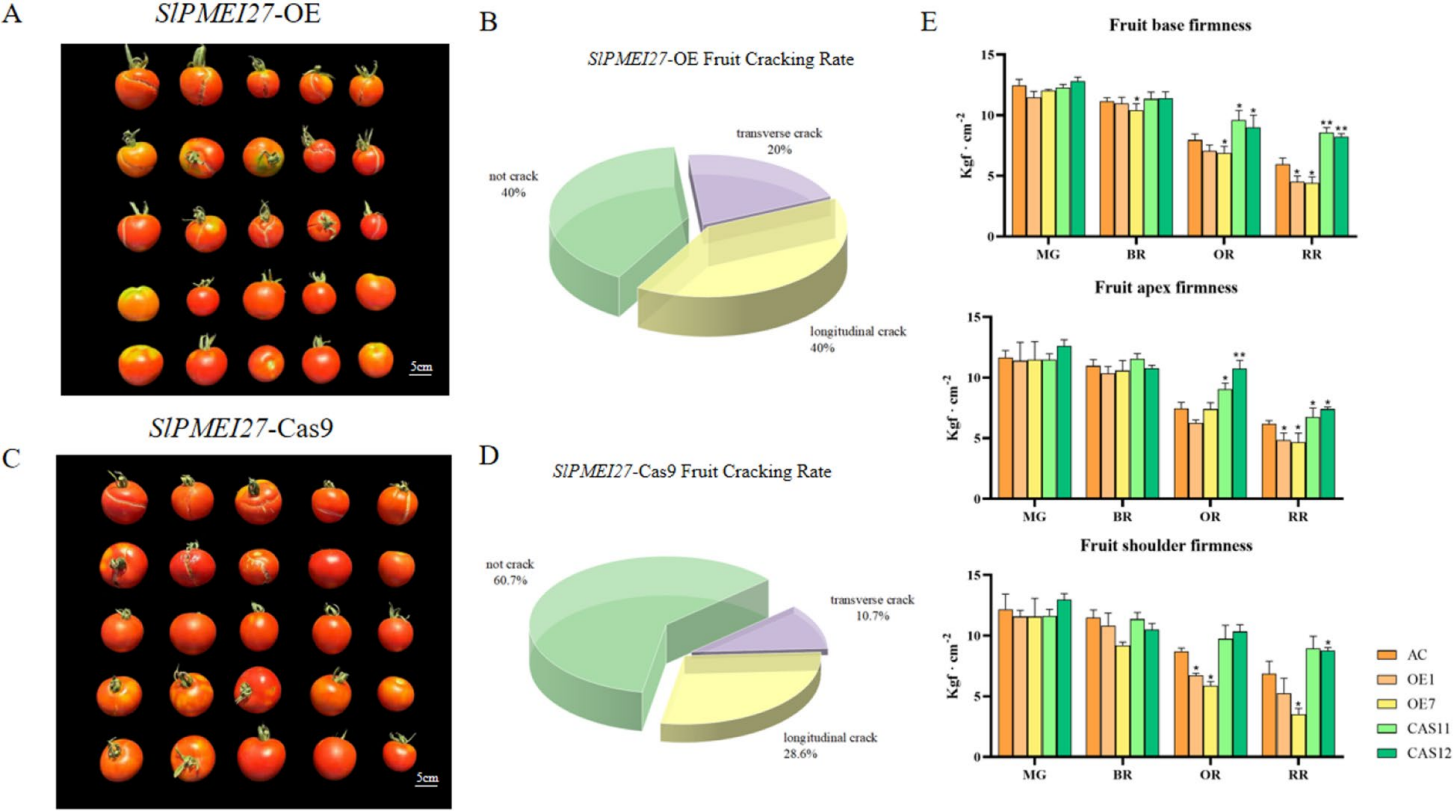
Fruit cracking ratio and firmness analysis in *SlPMEI27* transgenic lines at the RR stage. **A**,** B** Representative images and quantitative ratio of *SlPMEI27*-OE fruit cracking phenotypes at the RR stage. **C**, **D** Representative images and quantitative ratio of *slpmei27* fruit cracking phenotypes at the RR stage. **E** Fruit firmness at distinct fruit regions in RR stage tomatoes. * represents a significant difference, *P* < 0.05, ** represents a very significant difference,* P* < 0.01

At the RR stage, statistical analysis of cracking incidence indicated that OE lines exhibited the highest proportion of cracked fruits (60%), primarily displaying longitudinal cracks. In contrast, Cas9 lines demonstrated a significantly lower cracking rate (39.3%). (Fig. 2A-D) The cracking rate of WT was 44%, with longitudinal cracks being the predominant type (Fig. S1). Fruit firmness was assessed at three anatomical regions (shoulder, apex, and base) across four developmental stages. Cas9 fruits consistently displayed the highest firmness values, while OE fruits showed the lowest firmness values (Fig. 2E). Notably, firmness decreased progressively during ripening in all genotypes, with the most substantial reduction observed in OE fruits.

### Pectin methylesterification and cell wall remodeling

**Fig. 3:**
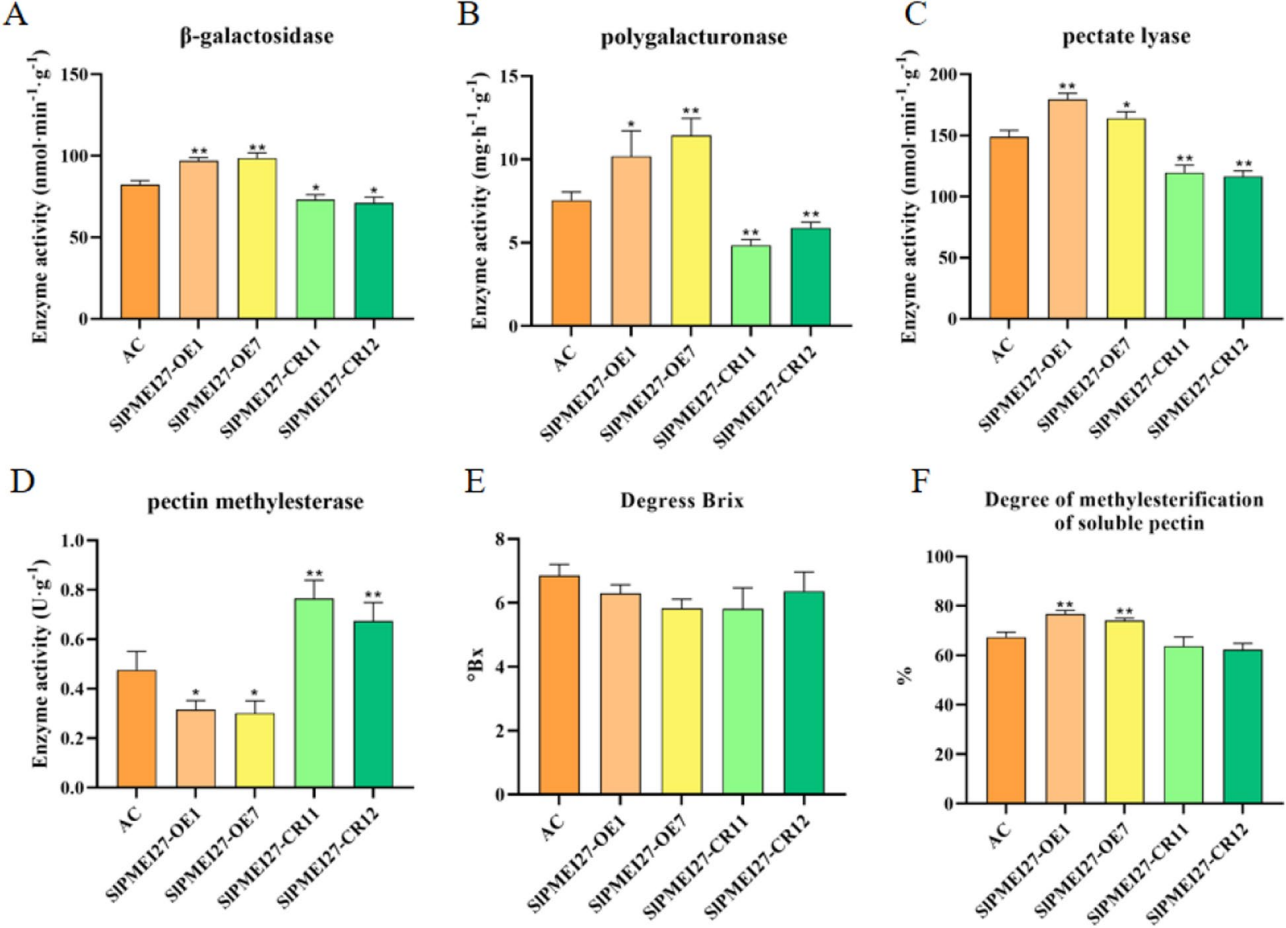
Biochemical characterization of cell wall modifications in *SlPMEI27* transgenic fruits. **A** β-Galactosidase (β-GAL) activity. **B** PG activity. **C** PL activity. D, PME activity. **E** Determination of fruit sugar content. **F** Degree of pectin methylesterification. * represents a significant difference, *P* < 0.05, ** represents a very significant difference,* P* < 0.01

To explore the underlying mechanisms, we analyzed the activity of key cell wall-modifying enzymes. Enzyme activity assays revealed a notable inverse relationship between *SlPMEI27* expression and PME activity, with PME levels reaching their peak in *slpmei27* fruits and their lowest value in *SlPMEI27*-OE fruits. Conversely, PL, β-GAL, and PG activities exhibited positive correlations with SlPMEI27 expression (Fig. 3A-D). Analysis of soluble solids content showed no significant differences in sugar accumulation among WT, *SlPMEI27-*OE, and *slpmei27* fruits (Fig. 3E). Pectin methylesterification status was confirmed through biochemical quantification (Fig. 3F). Chemical assays confirmed that *SlPMEI27-*OE fruits exhibited significantly higher methylesterification levels compared to WT and *slpmei27* fruits (*P* < 0.01).

**Fig. 4:**
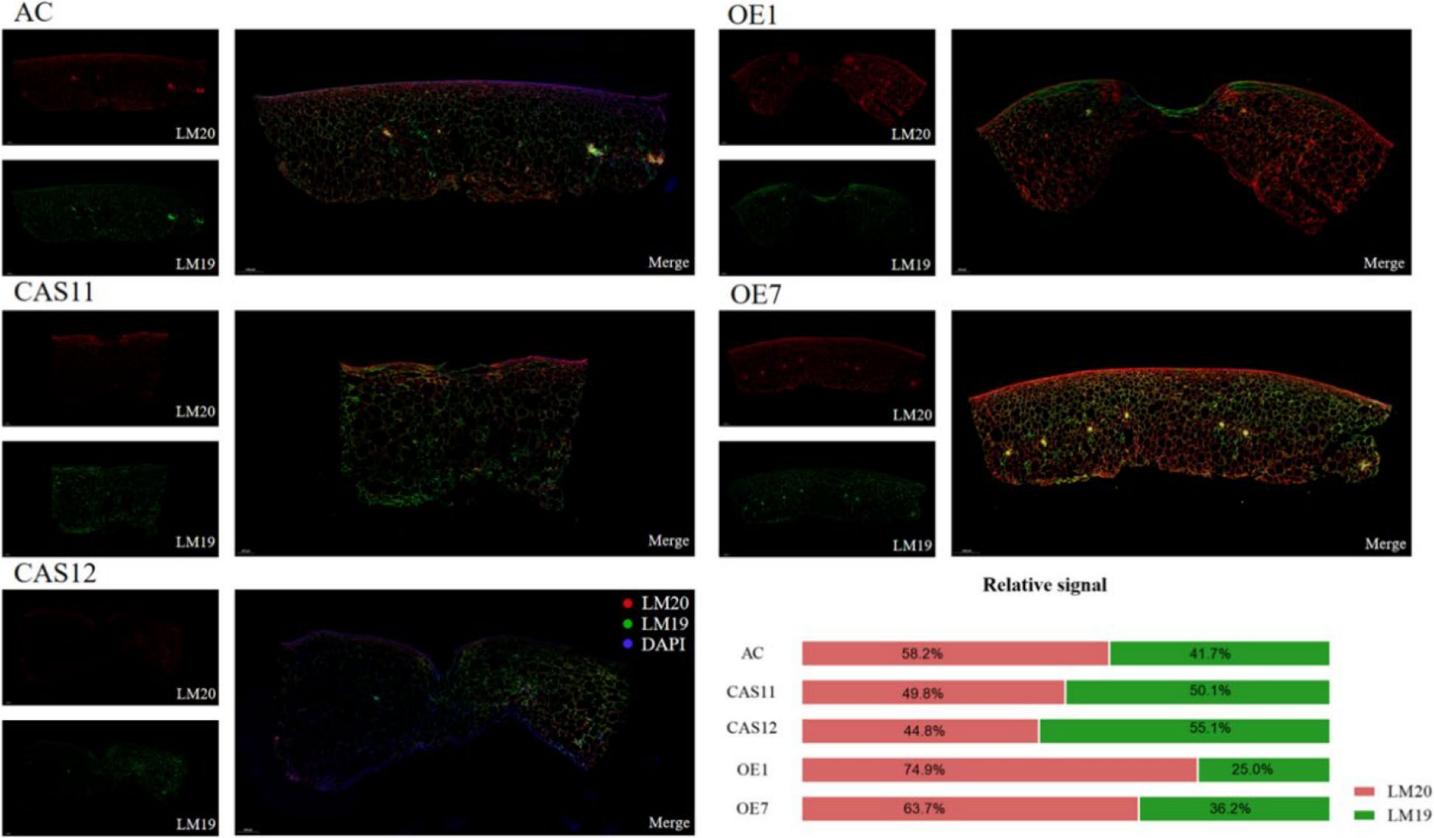
Immunofluorescence localization of pectin methylesterification status in tomato fruit cell walls. Immunolocalization of highly methylesterified HG (homogalacturonan) and de-esterified HG in *SlPMEI27*-OE, *slpmei27,* and AC fruit at RR stage. Monoclonal LM20 antibody probe recognizing highly methylesterifiedHG and LM19 probe recognizing de-esterified pectin were used to label tomato pericarp tissue. The graph on the right is the presentation of the fluorescence signal of each antibody after standardization using image J

Pectin methylesterification status was further examined by conducting an immunofluorescence assay with LM19 (which recognizes demethyl-esterified pectin) and LM20 (which recognizes highly methyl-esterified pectin). *SlPMEI27-*OE fruits displayed the strongest LM20 signal, indicating enhanced pectin methylesterification, while *slpmei27* fruits exhibited the highest LM19 intensity, indicating reduced methylesterification (Fig. 4).

## Microstructural and ultrastructural changes in fruit epidermis

**Fig. 5:**
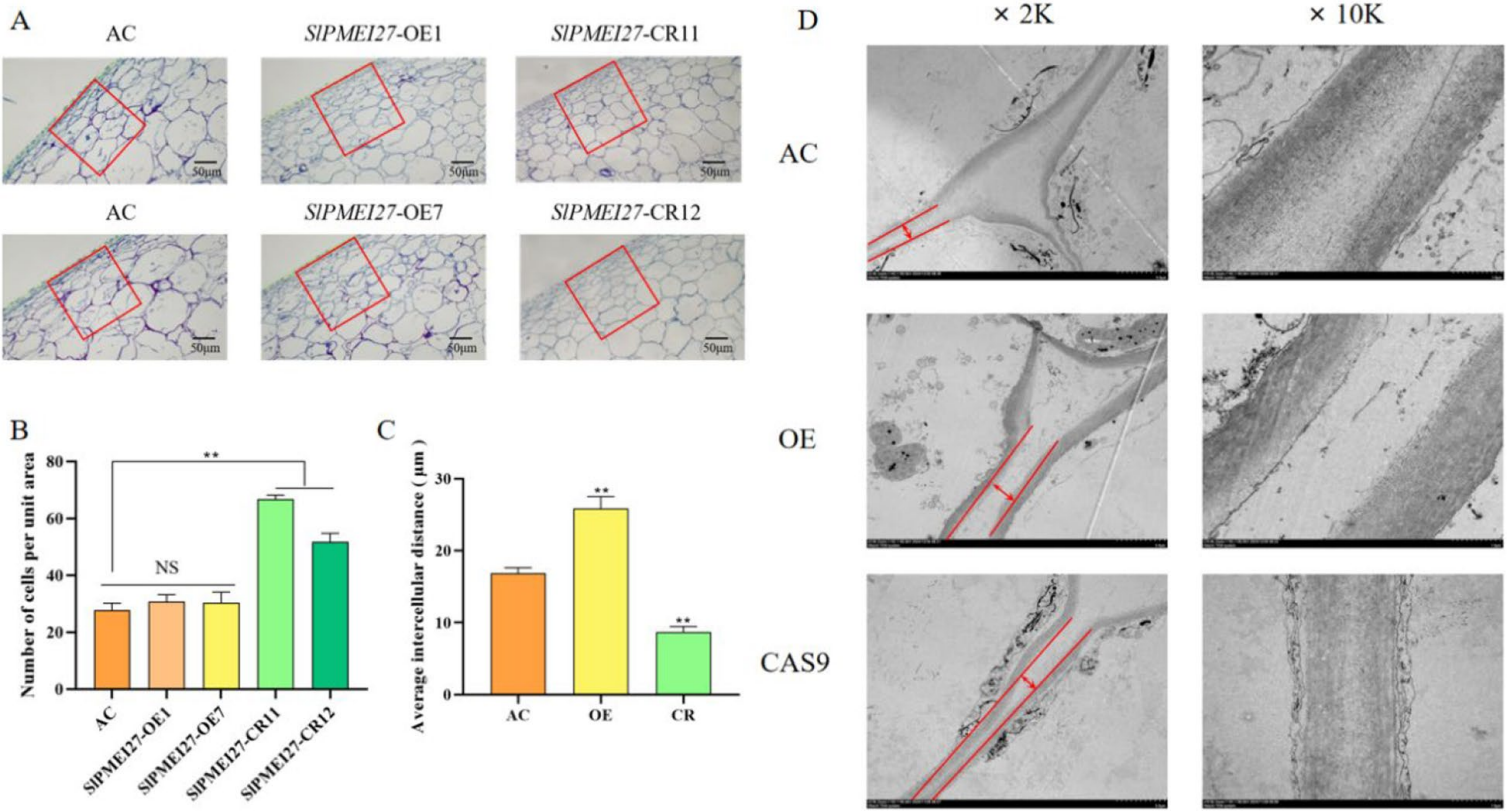
Histological and ultrastructural analysis of tomato fruit epidermis. **A** Paraffin sections of fruit epidermis. **B** Quantification of cell numbers per unit area (150 × 150 μm). **C** Measurements of intercellular space width. **D** TEM images of cell wall ultrastructure. * represents a significant difference, *P* < 0.05, ** represents a very significant difference,* P* < 0.01

Histological examination of fruit epidermis through paraffin sectioning revealed distinct cellular arrangements among the genotypes. *slpmei27* fruits displayed densely packed cells with the highest cell density per unit area, while *SlPMEI27-*OE and WT fruits demonstrated more irregular and loosely arranged cells (Fig. 5A, B). TEM further confirmed these findings: intercellular spaces were minimal in *slpmei27* fruits but notably expanded in *SlPMEI27-*OE fruits (Fig. 5C, D).

## Transcriptional regulation and protein interaction

**Fig. 6:**
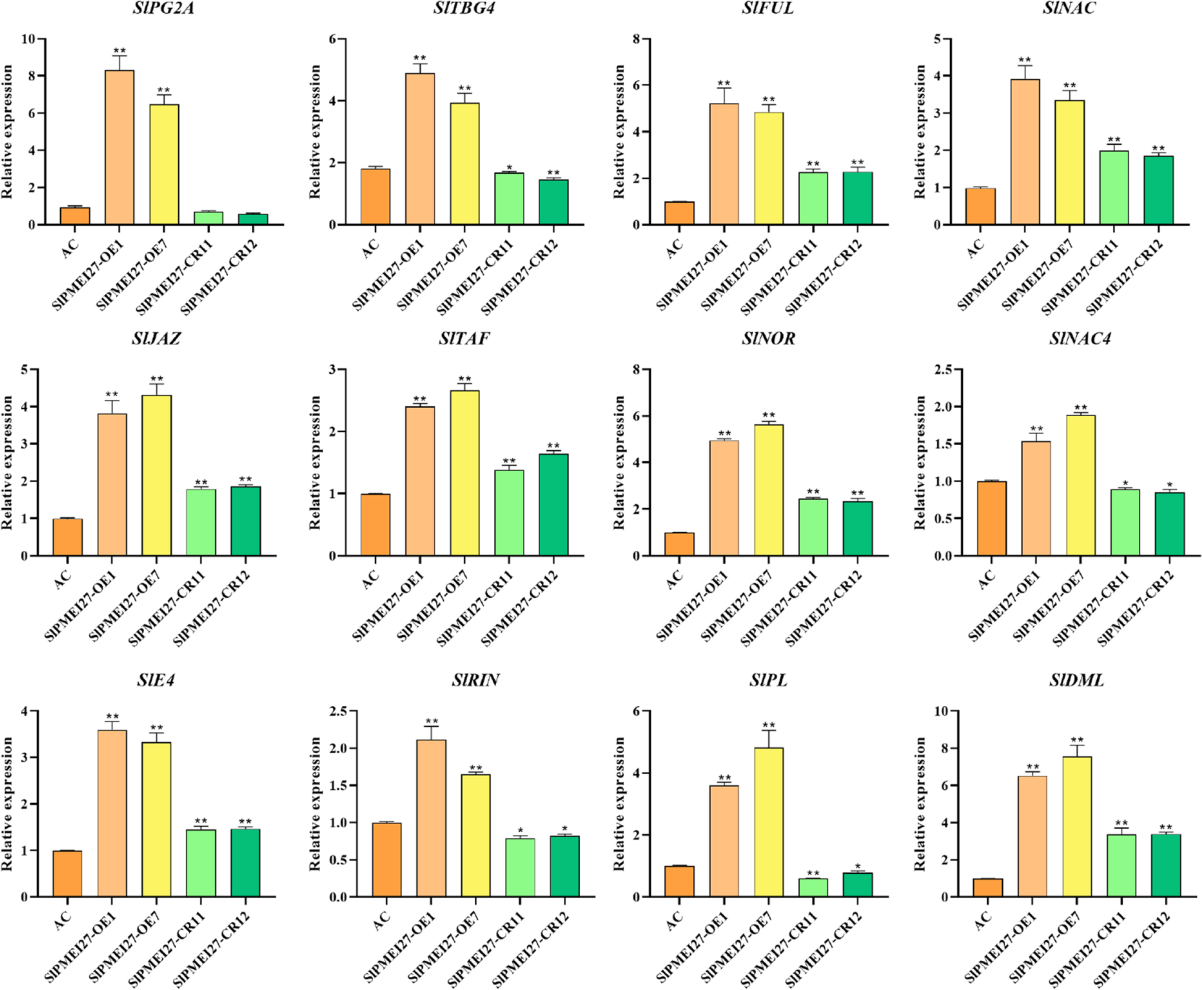
Expression profiles of ripening-related genes in *SlPMEI27* transgenic tomato fruits. * represents a significant difference, *P* < 0.05, ** represents a very significant difference,* P* < 0.01

To assess the broader impact of *SlPMEI27* manipulation on fruit ripening, we analyzed the expression of 12 ripening-related genes. In *SlPMEI27-*OE fruits, all tested genes were significantly upregulated compared to those in WT and *slpmei27* fruits (Fig. 6).

**Fig. 7:**
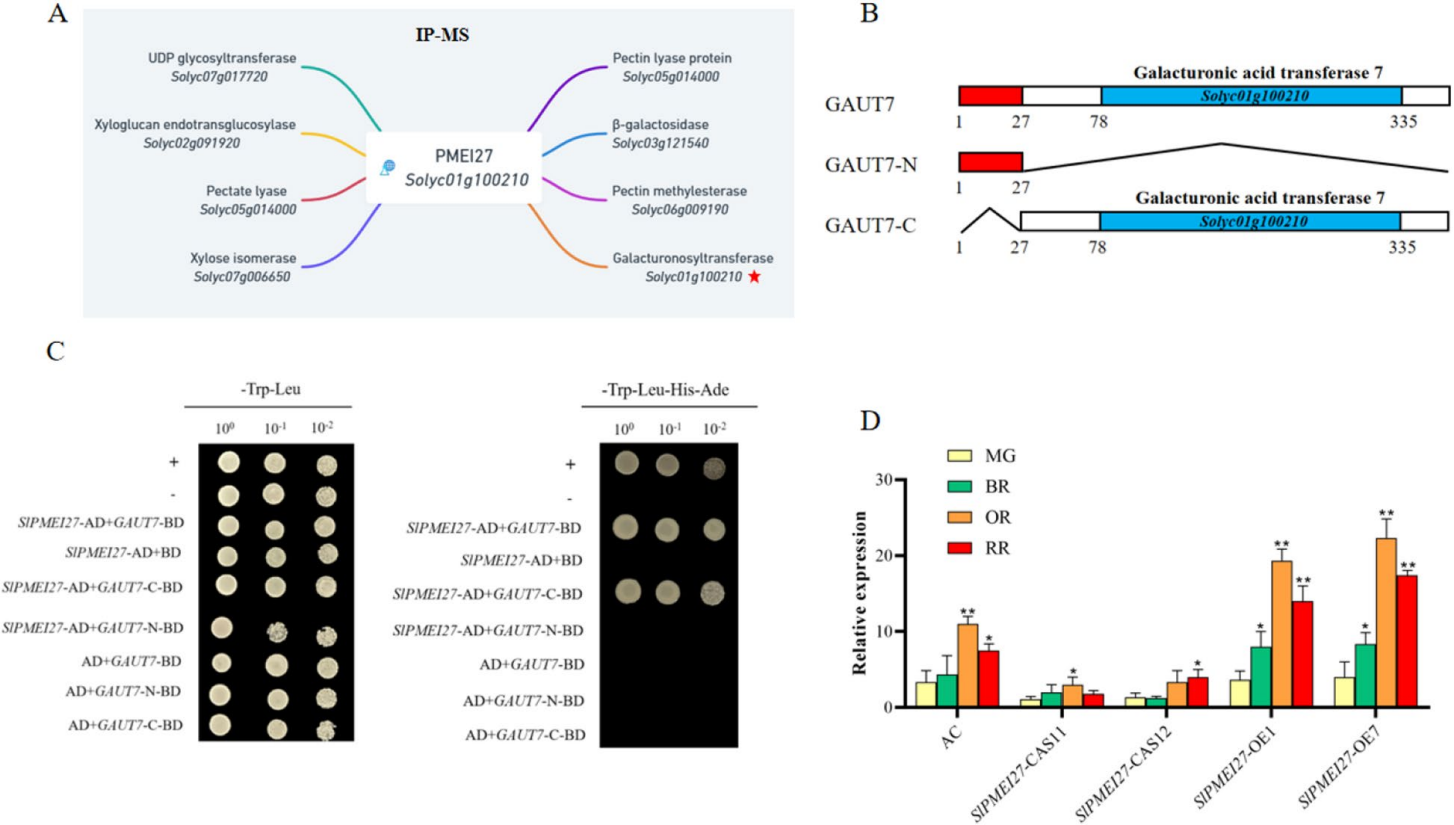
Identification and functional characterization of SlPMEI27-interacting proteins. **A** IP-MS analysis using SlPMEI27 as a bait. **B** Schematic representation of SlGAUT7 domain structure and truncated variants. N-terminal (1–27 aa) and C-terminal (28-end) fragments were generated for interaction studies. **C** Y2H assay demonstrating SlPMEI27-SlGAUT7 interaction. **D** Developmental expression profiles of SlGAUT7 in WT, *SlPMEI27-*OE, and *slpmei27* fruits across the ripening stages. * represents a significant difference, *P* < 0.05, ** represents a very significant difference,* P* < 0.01

To identify potential SlPMEI27 interacting partners, we conducted IP-MS using SlPMEI27 as a bait (Fig. 7A). Among the candidate interactors, we focused on galacturonosyltransferase (SlGAUT7) based on its established role in pectin biosynthesis. Structural analysis revealed that SlGAUT7 contains a glycosyltransferase domain, which prompted us to generate truncated variants: an N-terminal fragment (1–27 aa) and a C-terminal fragment (28-end) containing the catalytic domain (Fig. 7B). Y2H assays confirmed SlPMEI27-SlGAUT7 interaction, with domain mapping revealing specific binding to the C-terminal glycosyltransferase region (Fig. 7C). Developmental expression profiling showed coordinated regulation between these genes, with both SlGAUT7 and SlPMEI27 transcripts reaching the peak levels at the OR stage and showing highest abundance in *SlPMEI27-*OE fruits (Fig. 7D).

**Fig. 8:**
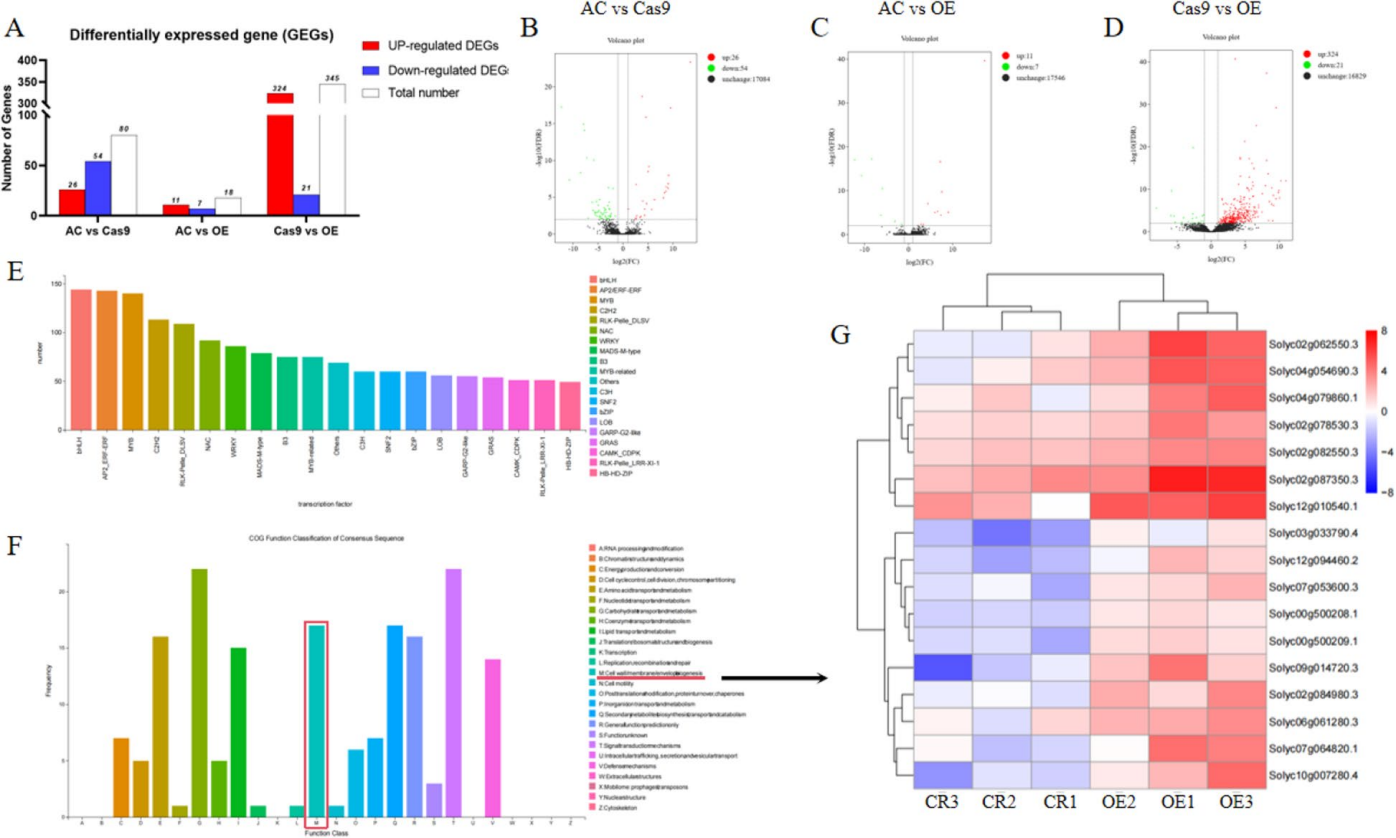
Transcriptomic landscape of *SlPMEI27*-regulated genes in red ripe (RR) tomato fruits. **A**-**D** Number of DEGs in pairwise comparisons (AC vs. Cas9, AC vs. OE, and OE vs. Cas9). **E** Transcription factor enrichment analysis of DEGs. **F** **G** Heatmap visualization of cell wall-related gene expression patterns

RNA-seq analysis of WT, *SlPMEI27-*OE, and *slpmei27* fruits at the RR stage revealed alterations in genome-wide expression dependent on SlPMEI27 activity levels. Pairwise comparison analysis identified 80 DEGs between WT and *slpmei27* fruits, while *SlPMEI27-*OE versus *slpmei27* fruits showed the most substantial changes with 345 DEGs (324 upregulated and 21 downregulated). Notably, 93.9% of these DEGs were enhanced in *SlPMEI27-*OE fruits, indicating SlPMEI27 primarily functions as a transcriptional activator (Fig. 8A-D). Transcription factor enrichment analysis of DEGs revealed overrepresentation of several key transcription factor families involved in fruit development: bHLH, ERF, MYB, and C2H2 (Fig. 8E). Functional classification demonstrated significant enrichment of cell wall-related genes. Heatmap visualization showed consistent upregulation of these cell wall modifiers in *SlPMEI27-*OE fruits compared to their downregulation in *slpmei27* fruits (Fig. 8F, G).

We validated these findings by qPCR analysis of 12 representative genes involved in pectin modification and transcriptional regulation (Fig. S3). All 12 genes exhibited expression patterns consistent with the RNA-seq data.

## Discussion

Fruit cracking poses a significant challenge in horticultural production, with current research primarily addressing external factors such as growth regulators and climatic conditions (Khadivi-Khub 2015). Previous studies have demonstrated that exogenous calcium application can reduce cracking incidence in citrus by inhibiting pectin and cellulose degradation, thereby enhancing fruit firmness and cell wall thickness (Tam, Singh, and Behboudian 2012). Additionally, gibberellic acid (GA3) treatments mitigate fruit cracking in pomegranate (*Punica granatum* L.) by maintaining cuticle integrity and improving cellular connectivity (Correia et al. 2020; Zahedi et al. 2022). Environmental factors, particularly temperature and precipitation patterns during fruit maturation, serve as critical determinants of cracking susceptibility in sweet cherry (Correia et al. 2018). Nevertheless, the intrinsic genetic mechanisms underlying fruit cracking remain inadequately understood. The present study provides novel insights into the molecular regulation of tomato fruit cracking through the characterization of *SlPMEI27*. The spatiotemporal expression pattern of *SlPMEI27*, peaking at the OR stage in fruit tissues, aligns with the developmental window of cracking susceptibility. Genetic evidence from *SlPMEI27-*OE1/7 and *slpmei27-*CR11/12 lines definitively establishes *SlPMEI27* as a master regulator of fruit cracking, with *SlPMEI27-*OE1/7 fruits showing premature cracking from the BR stage, while *slpmei27-*CR11/12 fruits maintain their structural integrity. This developmental stage-specific effect emphasizes the importance of temporal regulation in cell wall remodeling processes, as premature or delayed pectin modification disrupts the delicate balance between rigidity and flexibility required for stress tolerance.

At the mechanistic level, we demonstrate that SlPMEI27 functions as a molecular switch controlling pectin methylesterification homeostasis. Biochemical and cytological analyses revealed that *SlPMEI27* overexpression resulted in highly methyl-esterified pectin (approximately 70% vs. approximately 46% in Cas9 fruits) through the suppression of PME activity, leading to reduced calcium-mediated crosslinking and weakened cell adhesion. In contrast, *slpmei27* lines exhibited PME-catalyzed removal of methylester groups from highly methyl-esterified HGA, forming demethyl-esterified HGA (DMHG). DMHG then crosslinks with Ca^2+^ to form “egg-box” structures, enhancing cell wall strength and rigidity, thereby maintaining intercellular adhesion. These changes were visually apparent in TEM images, which showed expanded intercellular spaces in *SlPMEI27-*OE fruits vs. tightly packed cells in *slpmei27* lines. The consequent reduction in tissue firmness (*slpmei27-*CR11/12 fruits were approximately 1.8-fold firmer than *SlPMEI27-*OE1/7 fruits at the RR stage) directly contributes to mechanical vulnerability to cracking. These biochemical changes correlated quantitatively with cracking severity, establishing a predictive relationship between pectin methylesterification degree and cracking risk. This regulatory mechanism is consistent with the findings of Liu et al., where *SlBES1* transcriptionally repressed *PMEU1* to regulate pectin demethylesterification, thereby promoting tomato fruit softening (Liu et al. 2021).

The structural integrity of pectin in fruits is coordinately regulated by multiple enzymes, and modifications in pectin-modifying enzymes significantly influence fruit cracking susceptibility. For example, suppression of PL activity in strawberry (*Fragaria* × *ananassa* Duch.) maintains pectin content in the cell wall, enhances fruit firmness, and reduces cracking incidence (Santiago-Doménech et al. 2008). Similarly, the downregulation of PME and PG gene expression in grape berry skins during development decreases PME and PG activities, increases native pectin content, and consequently lowers fruit cracking rates (Martins et al. 2020). These findings collectively highlight the conserved role of pectin metabolism in cracking across diverse fruit species, though the specific regulatory nodes may vary.

Our study identified a novel functional interaction between SlPMEI27 and SlGAUT7, revealing a dual regulatory mechanism that concurrently governs pectin metabolism. SlPMEI27 modulates pectin modification through PME inhibition while influencing pectin biosynthesis through GAUT7 regulation. This sophisticated dual-targeting mechanism represents an evolutionarily optimized strategy for precisely coordinating cell wall dynamics during critical phases of fruit expansion. The observed synchronous expression peaks of both genes at the OR stage substantiate their functional synergy, while the dramatic cell wall modifications in our experimental systems demonstrate the physiological significance of this coordinated regulation.

The regulation of fruit cell wall dynamics involves an intricate genetic network. Transcriptomic approaches have emerged as effective tools for systematically investigating the molecular mechanisms underlying fruit cracking (Chen et al. 2019; Jiang et al. 2019; Niu et al. 2020; Zhu et al. 2020). Our transcriptomic profiling revealed the extensive influence of SlPMEI27 on gene regulatory networks, with 324 of 345 DEGs (93.9%) exhibiting upregulation in OE fruits. Significant enrichment was observed in cell wall-modifying enzymes (pectinases and expansins) and transcription factors (bHLH, ERF), many of which demonstrate established roles in fruit maturation and cell wall remodeling (Taylor-Teeples et al. 2015; Lee et al. 2016; Sakamoto et al. 2018; Wang et al. 2019; Wessels et al. 2019; Chen et al. 2019). The coordinated upregulation of cell wall-modifying and ripening-related genes in OE lines indicates that SlPMEI27 accelerates both the ripening program and cell wall degradation and restructuring. This dual effect likely contributes to the premature onset of cracking observed in transgenic fruits.

**Fig. 9:**
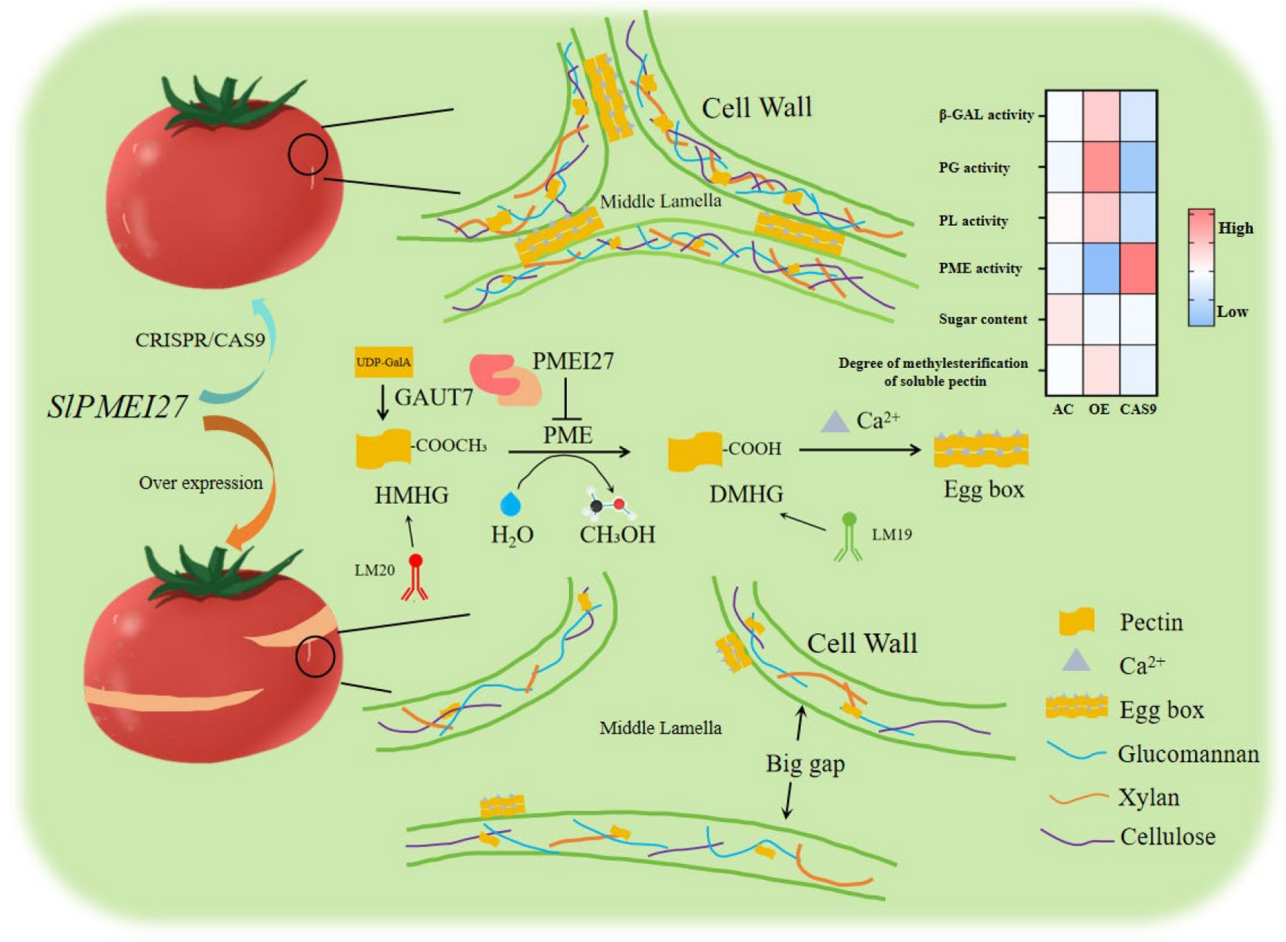
The proposed working model

The proposed working model (Fig. 9) synthesizes these findings, providing a comprehensive framework for understanding fruit cracking at the molecular level.

## Conclusion

This study demonstrates that SlPMEI27 plays a crucial role in regulating tomato fruit cracking by coordinating pectin methylesterification and cell wall remodeling. Genetic evidence indicates that *SlPMEI27* overexpression promotes cracking through PME suppression (70% methylesterification), while CRISPR knockout enhances integrity through PME-mediated de-methylesterification (46%) and strengthened wall cohesion. The present research reveals a novel SlPMEI27-SlGAUT7 interaction that synchronizes pectin biosynthesis and modification during ripening. Transcriptomic analysis further revealed that the regulatory network of SlPMEI27 involves cell wall enzymes (PL/PG/EXP) and ripening-related transcription factors (bHLH/ERF/MYB). These findings not only advance our understanding of fruit cracking mechanisms but also provide potential molecular targets for breeding crack-resistant tomato varieties while maintaining ripening quality.

## Materials and methods

### Plant materials and growth conditions

For the functional characterization of *SlPMEI27*, tomato (*Solanum lycopersicum)* seeds of the Ailsa Craig (AC) variety were used, which were provided by the Horticultural Biotechnology Team of Northeast Agricultural University. The AC seeds were moistened with distilled water, placed in plastic Petri dishes, and incubated in the dark at 28℃. After germination, the seeds were transplanted into a pre-mixed soil substrate (flower soil: vermiculite = 2:1) and transferred to a plant growth chamber under controlled conditions (25℃, 80% humidity, 16 h light/8 h dark cycle).

### Tomato genetic transformation

For genetic transformation, tomato seeds were initially soaked in distilled water overnight. Under sterile conditions, the seeds were surface-sterilized by immersion in 75% ethanol for 2–3 min, followed by three washes with sterile distilled water under agitation. Subsequently, the seeds were treated with a sodium hypochlorite solution (2–3% available chlorine) for 15 min, rinsed three times with sterile distilled water, and distributed evenly onto prepared 1/2 MS solid medium in culture bottles. Following 2–3 days of incubation in the dark, the germinated seeds were transferred to a growth chamber (25℃, 80% humidity, 16 h light/8 h dark cycle). Complete plants were generated using the *Agrobacterium*-mediated method after preculture, co-culture, screening culture, and root culture.

### Methods for determining fruit traits

WT, *SlPMEI27*-OE, and *slpmei27* fruits were selected at four developmental stages: Mature Green (MG), Breaker (BR), Orange Ripening (OR), and Red Ripening (RR), ensuring uniform size and growth conditions. Fruit firmness was measured at the shoulder, apex, and base by using a fruit hardness tester (Model #FHT-05, Guangzhou Landtek Instruments Co., Ltd.), with three biological replicates. For field evaluations, RR stage fruits were harvested randomly from WT, *SlPMEI27*-OE, and *slpmei27* plants. A minimum sample size of *n* = 25 fruits per replicate group was used to ensure statistical power, and the experiment was conducted in triplicate. The fruit cracking rate was calculated as follows:

$$\mathrm{Cracking}\;\mathrm{rate}\left(\%\right)=\frac{\mathrm{Number}\;\mathrm{of}\;\mathrm{cracked}\;\mathrm{fruits}}{\mathrm{Total}\;\mathrm{fruits}\;\mathrm{assessed}}\times100\%$$

### Determination of physiological and biochemical indices

RR stage tomato fruits exhibiting uniform growth were selected from *SlPMEI27*-OE, *slpmei27* and WT lines. Fruit sugar content was determined using a digital refractometer (Model #SW-32A, Suwei Technology Co.). For each measurement, fruits were halved, and 2 mL of fruit juice was pipetted into the sample chamber. After recording the data, the chamber was rinsed with distilled water and dried with a filter paper before subsequent measurements. Three biological replicates were conducted. To assess pectin-modifying enzyme activities, 0.1 g of pericarp tissue (including both exocarp and mesocarp) from RR stage fruits was collected and homogenized in 1.5 mL centrifuge tubes. Commercial assay kits (PME-2-G for PME, PL-2-G for PL, PG-1-G for PG, and GALB-2-Y for β-galactosidase; Suzhou Keming Biotechnology Co., Ltd.) were used in accordance with the manufacturer’s instructions. The degree of methylesterification of soluble pectin was determined following the method reported by Liu et al. (Liu et al. 2021).

### Paraffin sectioning

Fruit peel samples from AC (WT), *SlPMEI27*-OE, and *slpmei27* lines were harvested (RR stage), sectioned into 1 cm2 pieces, and fixed in FAA (Formalin-Acetic Acid-Alcohol) for 24 h. Fixed tissues were dehydrated through a graded ethanol series (70%, 85%, 95%, and 100% ethanol, 2 h per step), followed by clearing in a xylene-ethanol gradient (2:1, 1:1, 1:2, and pure xylene, 2 h each). Samples were infiltrated with paraffin wax by adding wax chips until saturation, incubated at 37℃ overnight, and embedded in melted paraffin. After solidification, the blocks were trimmed and sectioned (10–15 μm thickness) using a rotary microtome. The sections were mounted on glass slides coated with Mayer’s adhesive, dried at 37℃, and deparaffinized in xylene (3 times × 5 min each). Rehydration was performed through a descending ethanol series, followed by staining with 0.1% toluidine blue (30 min). Slides were rinsed, dehydrated, cleared in xylene (3 times × 2 min each), and mounted with neutral resin under coverslips. Images were captured using a light microscope with consistent magnification.

### Transmission electron microscopy (TEM) sample preparation

Fruit tissues (RR stage) were trimmed into small strips (3 mm in length and 1 mm in width) and fixed with 2.5% glutaraldehyde in phosphate buffer (pH 6.8) for at least 2 h. After rinsing three times with 0.1 M phosphate buffer (15 min each), the samples were post-fixed with 1% osmium tetroxide, followed by additional buffer rinses. Dehydration was performed using a graded ethanol series (50%, 90%, and 100%) and pure acetone, with all steps performed at 4℃. The samples were subsequently infiltrated with a mixture of acetone and embedding resin at varying ratios (1:1, 1:2, and 1:3) at room temperature (RT), embedded the next day, and polymerized. Ultrathin Sects. (50–60 nm) were prepared using an ultramicrotome, double-stained with uranyl acetate and lead citrate, and examined under a transmission electron microscope for imaging.

### Immunofluorescence staining

After dewaxing and antigen retrieval, endogenous peroxidases were blocked with 3% H₂O₂ (25 min, RT). The sections were blocked with bovine serum albumin and incubated with the first primary antibodies, followed by incubation with HRP-conjugated secondary antibodies. Tyramide signal amplification was performed using Cy3- or FITC-conjugated tyramine. Antibody complexes were removed by microwave treatment in EDTA buffer. The second primary antibodies were applied, detected with fluorescent secondary antibodies, and counterstained with DAPI. After autofluorescence quenching, the slides were mounted and imaged as mentioned above. Immunofluorescence co-localization analysis was conducted using monoclonal antibodies LM19 (AS18 4191, Agrisera) targeting de-esterified HGA and LM20 (AS18 4193, Agrisera) specific to highly methyl-esterified pectin epitopes.

### Immunoprecipitation-mass spectrometry (IP-MS) and yeast two-hybrid assay

Protein gel samples were processed and stored at −20℃ until liquid chromatography-tandem mass spectrometry (LC–MS/MS) analysis. MS data were acquired using a Q Exactive HF-X mass spectrometer coupled with an EASY-nLC 1200 nanoflow HPLC system. Raw data were processed using MaxQuant (v1.6.6) with the Andromeda search algorithm against the UniProt tomato proteome database (release 2020–12–21; 34,652 protein entries). The yeast two-hybrid (Y2H) assay was performed using Y2HGold yeast competent cells (Coolaber, #CC309). Briefly, thawed competent cells were combined with 1 µg each of SlPMEI27-AD and GAUT7-BD plasmids, 10 µL heat-denatured carrier DNA, and 500 µL PEG/LiAc, followed by incubation at 30℃ for 30 min (with mid-point mixing). Cells were heat-shocked at 42℃ for 15 min, centrifuged (5000 rpm, 40 s), and resuspended in 400 µL ddH₂O. Following another round of centrifugation, the cells were resuspended in 50 µL ddH₂O and plated on SD/-Leu/-Trp plates. Colonies (2–3 mm) were selected after 48–96 h at 28 °C, resuspended in sterile water to OD_600_ = 0.2, and serially diluted (10 ×, 100 ×, and 1000 ×). For interaction testing, 10 µL of each dilution was spotted onto SD/-Leu/-Trp (control) and SD/-Leu/-Trp/-His/-Ade (selection) plates.

### Transcriptome analysis and gene expression validation

Fruits at the RR stage were collected and sent to Biomarker Technologies Corporation for RNA sequencing (RNA-seq) analysis, with three biological replicates per group. Transcriptomic changes between WT and SlPMEI27 transgenic lines were analyzed following our previously published methodology (Meng et al. 2022). To validate the RNA-seq results, 10 ripening-related genes were selected, and their expression patterns were examined in WT and T_2_ generation transgenic fruits using qRT-PCR (primers listed in Table S1). Additionally, differentially expressed genes (DEGs) associated with fruit ripening and cell wall metabolism were identified from the transcriptome data, and their expression profiles were analyzed (primers listed in Table S2).

## Supplementary Information

Supplementary Material 1.

## Data Availability

All relevant data are within the paper.

## Abbreviations

PMEs: Pectin methylesterase
PL: Pectate/pectin lyases
PG: Polygalacturonases
β-Gal: β-Galactosidases
HGA: Homogalacturonan
GAUTs: Galacturonosyltransferases
PMEIs: Pectin methylesterase inhibitors
PGs: Polygalacturonases
PLs: Pectate/pectin lyases
SlPMEI27-OE: SlPMEI27-overexpressing
WT: Wild-type
slpmei27: SlPMEI27-Cas9
MG: Mature Green
BR: Breaker
OR: Orange Ripening
RR: Red Ripening
Cas9: CRISPR-associated protein 9
qPCR: Quantitative Real-Time Polymerase Chain Reaction
OE: Overexpressing
HG: Homogalacturonan
IP-MS: Immunoprecipitation-Mass Spectrometry
SlGAUT7: Galacturonosyltransferase
Y2H: Yeast Two-Hybrid System
RNA-seq: RNA sequencing
DEGs: Differentially Expressed Genes
GA3: Gibberellic acid
CR: CRISPR
DMHG: Demethylesterified HG
HMHG: Highly methylesterified HG
TEM: Transmission Electron Microscope
AC: Ailsa Craig
MS: Murashige and Skoog
FAA: Formalin-Acetic Acid-Alcohol
RT: Room temperature
HRP-conjugated: Horseradish Peroxidase-conjugated
Cy3: Cyanine3
FITC-conjugated tyramine: Fluorescein Isothiocyanate-conjugated tyramine
EDTA: Ethylenediaminetetraacetic Acid
LC–MS/MS: Liquid chromatography-tandem mass spectrometry
MS: Mass spectrometry
HPLC: High Performance Liquid Chromatography
PEG/LiAc: Polyethylene Glycol/Lithium Acetate
ddH₂O: Double distilled water
RNA-seq: RNA sequencing
