# Supplementary material for: Genetic control of tomato fruit cracking through SlPMEI27-dependent pectin modification
Source: Mol Hortic. 2026 May 11;6:34. doi: 10.1186/s43897-025-00219-2 (PMC13159324; doi:10.1186/s43897-025-00219-2)
Supplement: Supplementary file 1 — Supplementary Material 1. [file 43897_2025_219_MOESM1_ESM.docx]

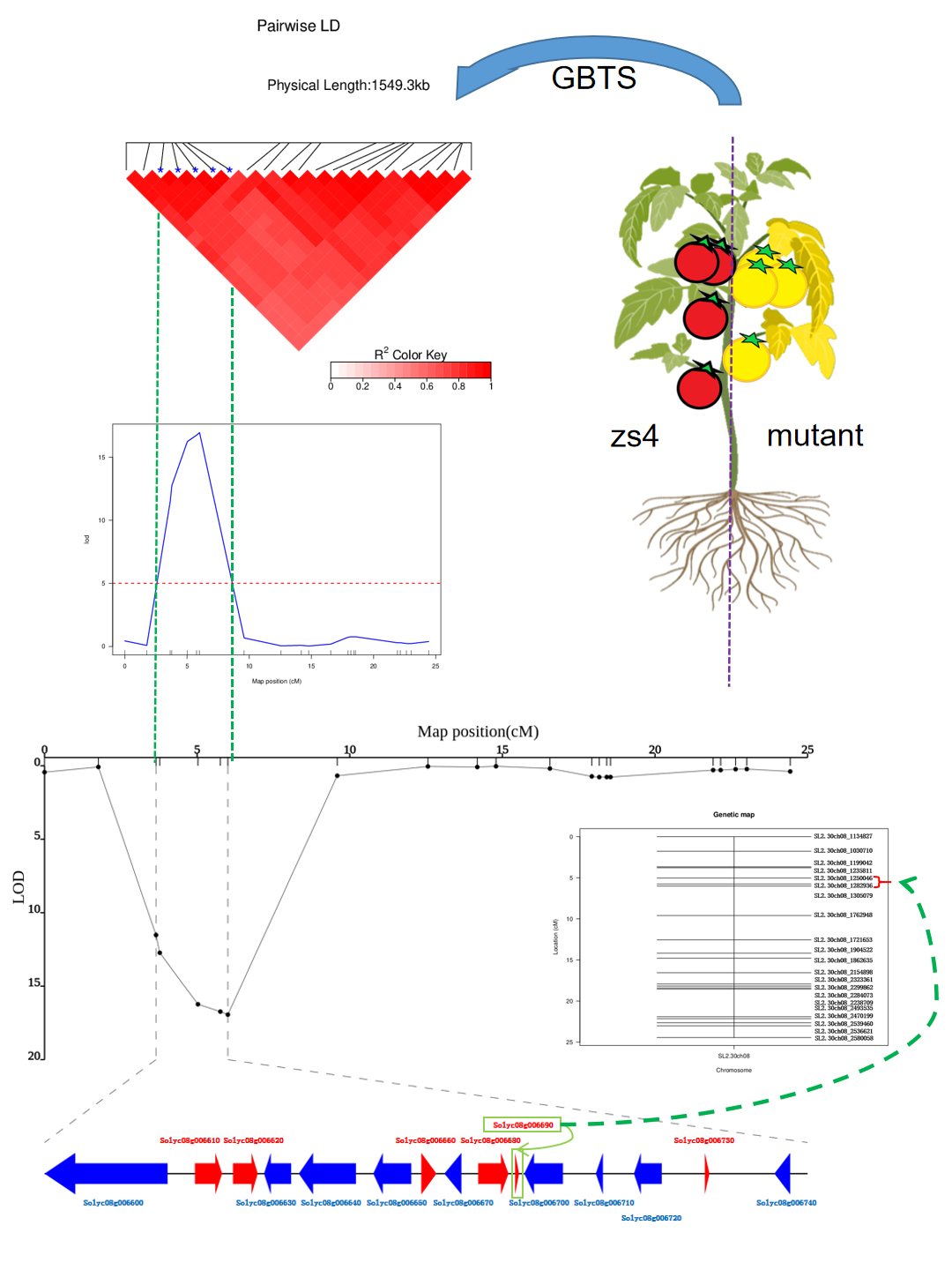


Fig. S1 Fine-mapping of the key candidate gene.


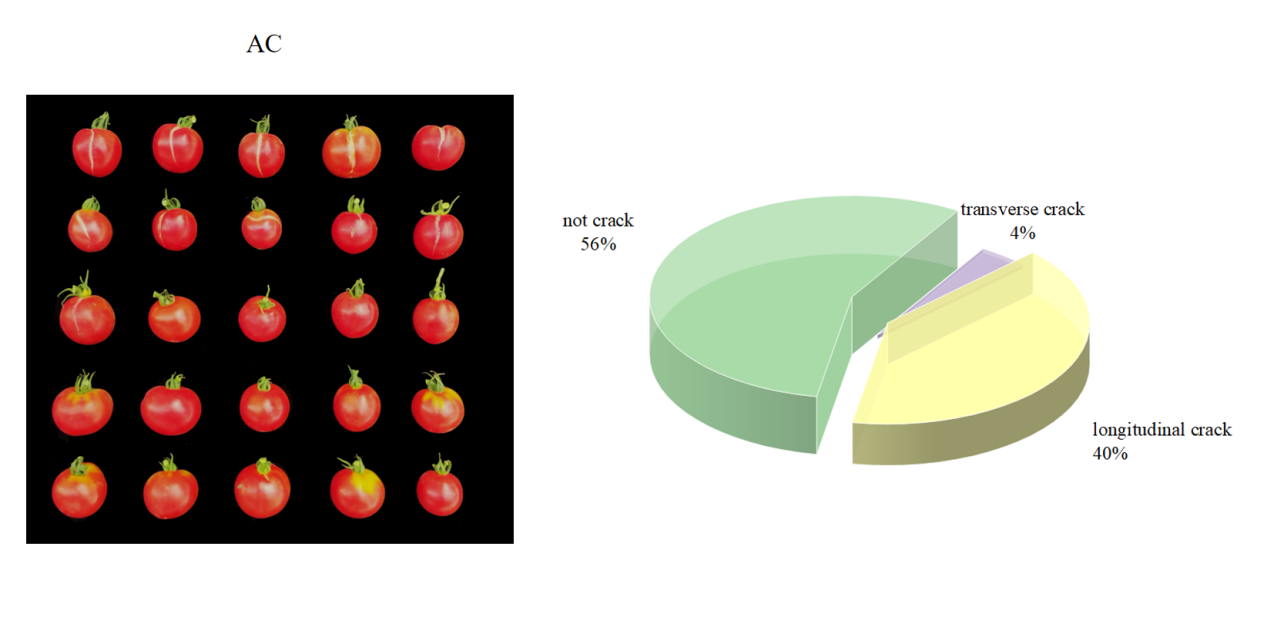


Fig. S2 Fruit cracking ratio in RR stage tomato fruits.


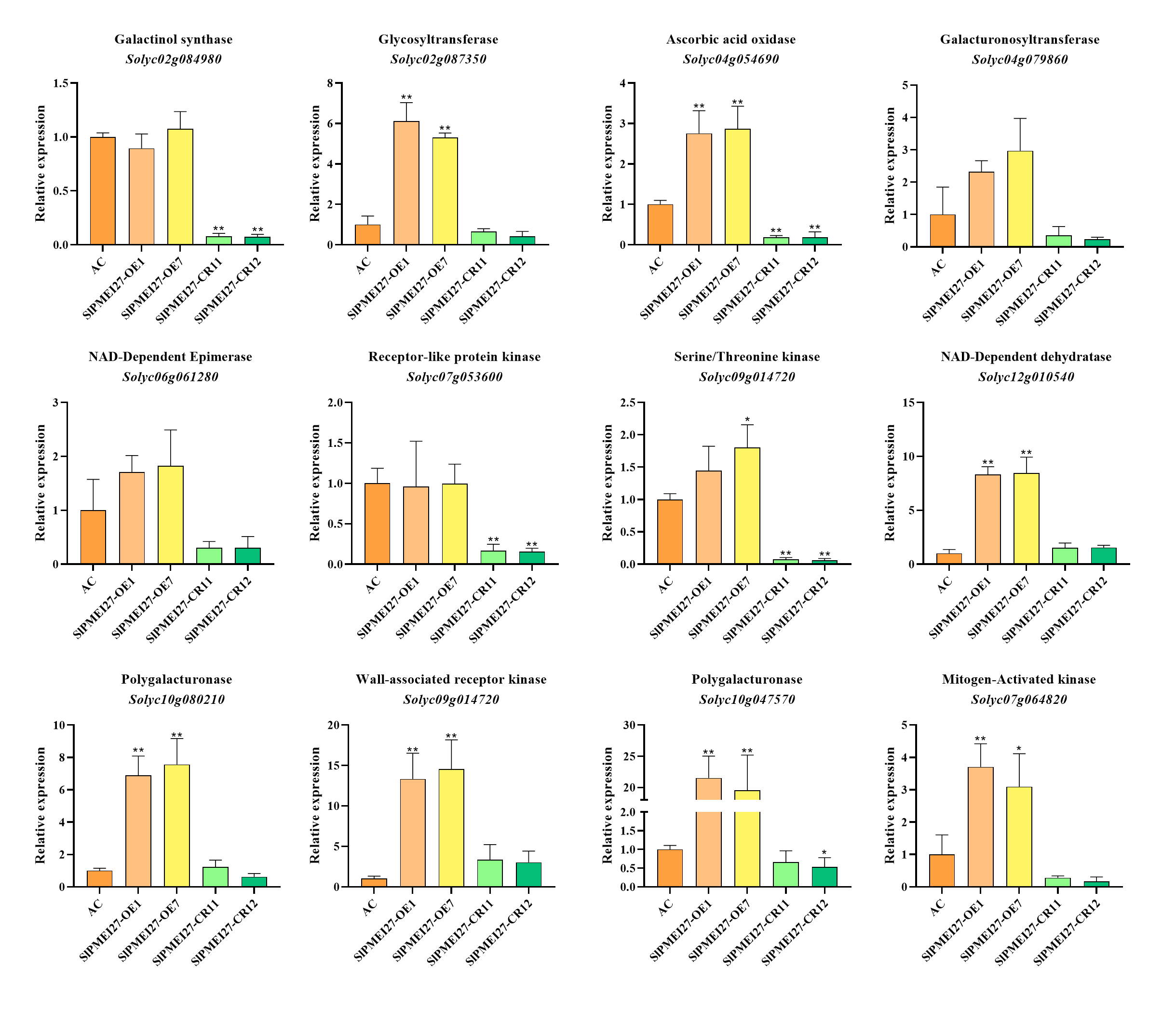


Fig. S3 Validation of RNA-seq results by qPCR analysis of selected genes. * represents a significant difference, *P* < 0.05, ** represents a very significant difference, *P* < 0.01.

**Table S1.** The sequences of primers for qRT-PCR analysis

| **Primer name** | **Primer sequences** |
| --- | --- |
| *SlPL*-F | 5’-GGAAATCCAATCGACGATTGTT-3’ |
| *SlPL*-F | 5’-CGTTTTTCCCAAATCCAATTGC-3’ |
| *SlACS4*-F | 5’-CTGAATTCACAAATGCGATTGC-3’ |
| *SlACS4*-R | 5’-CAGCCATTACTACACGTTTAGC-3’ |
| *SlTBG4*-F | 5’-GGTTCCGTCCTAATAAGCCTTA-3’ |
| *SlTBG4*-R | 5’-CAACCCATATTCATCGAGAGGA-3’ |
| *SlE4*-F | 5’-TGCCCGTATTCCAATCTCCT-3’ |
| *SlE4*-R | 5’-CTGCTTACAACCTCTGCCCC-3’ |
| *SlJA2*-F | 5’-GAAATTCAACCTCGATCGTCTG-3’ |
| *SlJA2*-R | 5’-CATATTGTTGTTGTCGCTGTGA-3’ |
| *SlTAF1*-F | 5’-CAGGAAAATGAACACTACCTGC-3’ |
| *SlTAF1*-R | 5’-TAAATGGAAGATCGAGGGTAGC-3’ |
| *SlPG2a*-F | 5’-CCAAAGGAATAGTATTCTCCTTCTC-3’ |
| *SlPG2a*-R | 5’-GTTTTTCCATCACCCTTAGCTC-3’ |
| *SlFUL1*-F | 5’-CATGAGTCCATTTCTGTGCTTC-3’ |
| *SlFUL1*-R | 5’-GATTTCCCACTGATTTTGCTGT-3’ |
| *SlDML2*-F | 5’-AGTACTCATGCCAAAGCCAAA-3’ |
| *SlDML2*-R | 5’-CCTATCTTCTTTTTACCGACTGGA-3’ |
| *SlRIN*-F | 5’-GCAAAGAGAAGAAATGGACTCC-3’ |
| *SlRIN*-R | 5’-GGGTTCCTTCAAGTGTACCATA-3’ |
| *SlNOR*-F | 5’-GAAAGTACGGATTCATCAACCG-3’ |
| *SlNOR*-R | 5’-TTGAGGTAGTGGACGATGA-3’ |
| *SlNAC4*-F | 5’-CACGTTTACAATGACTTCACGT-3’ |
| *SlNAC4*-R | 5’-TATCTTGTAGCGGTGACATCTC-3’ |

**Table S2.** The sequences of primers for qRT-PCR analysis

| **Primer name** | **Primer sequences** |
| --- | --- |
| Solyc02g084980-F | 5’-GTTGTTCACTACTGTGCGGC-3’ |
| Solyc02g084980-R | 5’-ACACCAGCCTCTGATAACGC-3’ |
| Solyc02g087350-F | 5’-AATCAACATGGGCTTGGTGGA-3’ |
| Solyc02g087350-R | 5’-CTTCGCTCTTGATCGCCCA-3’ |
| Solyc04g054690-F | 5’-AGCGCGAATTCGTCATTATCAA-3’ |
| Solyc04g054690-R | 5’-ATCAGCCCATGGTGTTCCAA-3’ |
| Solyc04g079860-F | 5’-CCACCGCAACAGTTCAGAGA-3’ |
| Solyc04g079860-R | 5’-GATGCGGAAGCAACGAAGTG-3’ |
| Solyc06g061280-F | 5’-GAGTCGTCGTCGTTGAAGGG-3’ |
| Solyc06g061280-R | 5’-GTGCGCATGCTTCCAATACA-3’ |
| Solyc07g053600-F | 5’-TTGGATTGGCCGTTGAGGTT-3’ |
| Solyc07g053600-R | 5’-ATGGATTCGACGCCACCTTT-3’ |
| Solyc09g014720-F | 5’-CCACCGCCCAAATTATCCCT-3’ |
| Solyc09g014720-R | 5’-AAGGCACAACCAGACCCAAT-3’ |
| Solyc12g010540-F | 5’-GCCCGGCTATGTTTCGGATA-3’ |
| Solyc12g010540-R | 5’-TACCCGAGTTCCTTTTGGGC-3’ |
| Solyc10g080210-F | 5’-CAGGGAGGATCTGGACAAGC-3’ |
| Solyc10g080210-R | 5’-CTTTGTTGCACTTGTGCCCT-3’ |
| Solyc09g014720-F | 5’-CCACCGCCCAAATTATCCCT-3’ |
| Solyc09g014720-R | 5’- AAGGCACAACCAGACCCAAT-3’ |
| Solyc10g047570-F | 5’-TGGACAAGGGGATTGTGCTT-3’ |
| Solyc10g047570-R | 5’-CCATCGGTGTTAGGGCTGTT-3’ |
| Solyc07g064820-F | 5’-ATTTTCCGGTACACCGGCTT-3’ |
| Solyc07g064820-R | 5’-GGGAATTGTGGCATATCCCCT-3’ |
